# Infarcted Leiomyoma With Hyaline Degeneration Mimicking Hematometra: A Case Report

**DOI:** 10.7759/cureus.112039

**Published:** 2026-07-04

**Authors:** Diksha Singla, Purnima Basumatary, Sunayna Lashkari, Shweta Patel, Sarat Manohar

**Affiliations:** 1 Obstetrics and Gynecology, All India Institute of Medical Sciences, Bhopal, IND; 2 Pathology, All India Institute of Medical Sciences, Bhopal, IND

**Keywords:** degeneration, hematometra, hyaline degeneration, leiomyoma, uterine leiomyoma

## Abstract

Uterine leiomyomas are common benign tumors that may undergo secondary degenerative changes, most frequently hyaline degeneration. Hemorrhagic (red) degeneration is uncommon outside pregnancy and can pose a diagnostic challenge. We report a 45-year-old hypertensive woman presenting with urinary frequency, dysuria, lower abdominal pain, and progressive abdominal distension. Examination revealed a uterus enlarged to 28-30-week size. Ultrasonography demonstrated an apparent intrauterine fluid collection, raising suspicion of hematometra or hydrometra. Cervical drainage was unsuccessful, with a possible diagnosis of organized collection. Magnetic resonance imaging showed a markedly enlarged uterus containing T1-hyperintense fluid suggestive of blood products, raising concern for malignancy. Total hysterectomy was performed. Frozen section excluded malignancy, and final histopathology confirmed an infarcted leiomyoma with hyaline degeneration and intratumoral hemorrhage. This case highlights a rare presentation of hemorrhagic degeneration in a non-pregnant woman and emphasizes the importance of histopathology in resolving the diagnostic uncertainty.

## Introduction

Uterine leiomyomas are the most common benign tumors and affect women predominantly during the reproductive and perimenopausal years [1]. Although many remain asymptomatic, larger fibroids may cause pain, pressure symptoms, or abnormal bleeding. Secondary degenerative changes in fibroids occur when tumor growth exceeds vascular supply [2]. Hyaline degeneration is the most common, followed by cystic, myxoid, and, rarely, hemorrhagic (red) degeneration [3]. Red degeneration represents hemorrhagic infarction due to venous thrombosis and is typically associated with pregnancy; occurrence in non-pregnant women is rare and may mimic malignancy or other pelvic pathology on imaging [4-6]. Degenerative changes may alter the usual imaging appearance of leiomyomas, occasionally mimicking other gynecological conditions such as ovarian neoplasms, adnexal masses, pyometra, or hematometra [7].

We report a rare case of an infarcted uterine leiomyoma with hyaline degeneration that clinically and radiologically mimicked hematometra. This case highlights the diagnostic challenges associated with unusual fibroid degeneration and radiologically mimic as hematometra. Misdiagnosis may lead to delays in appropriate management and surgical planning.

## Case presentation

**Figure 1:**
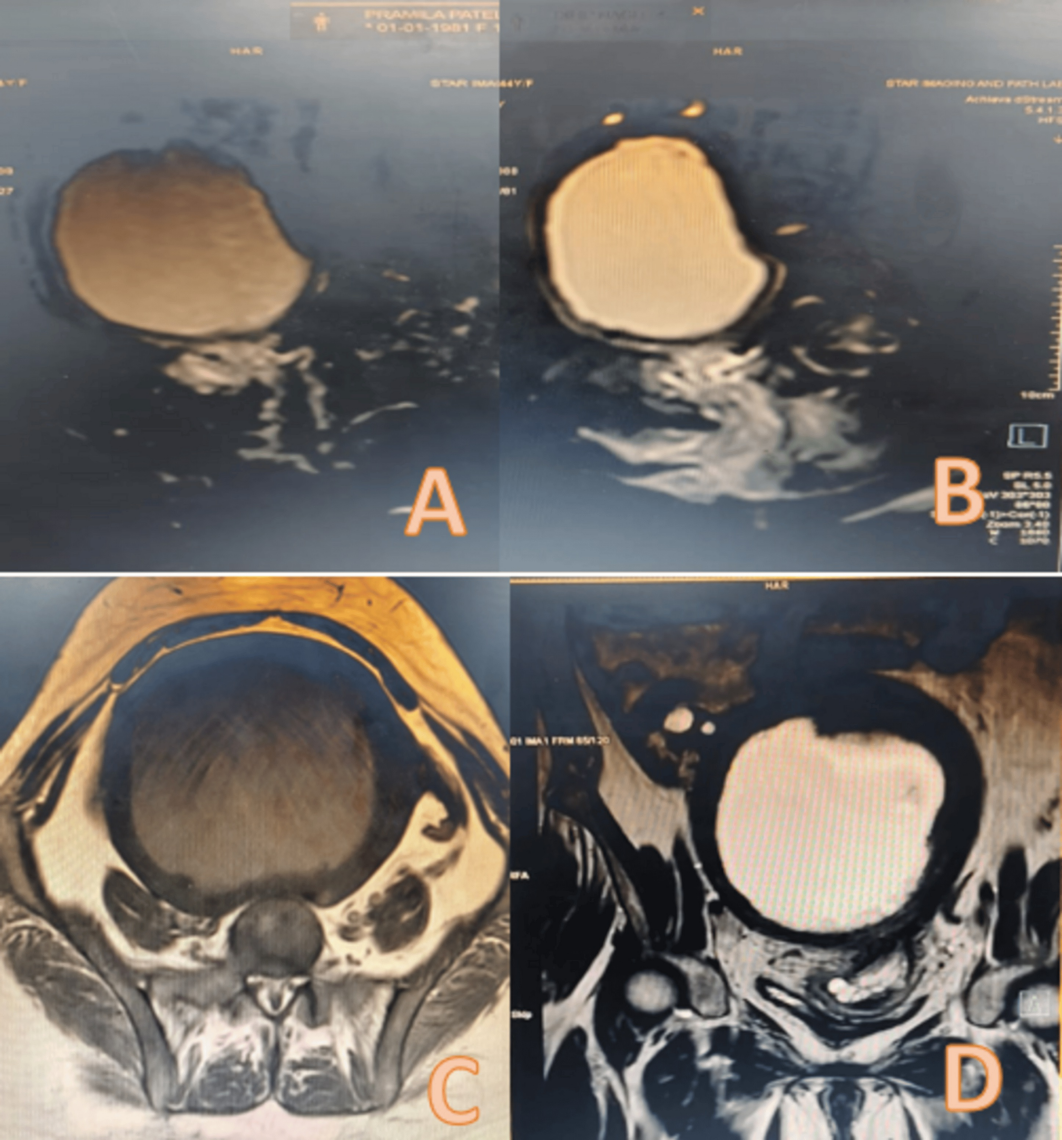
Diffusion-weighted MRI of the pelvic mass (A) Diffusion-weighted image (DWI, b=1000 s/mm²) demonstrating marked hyperintensity within the large pelvic mass. (B) Corresponding apparent diffusion coefficient (ADC) map showing low ADC signal (diffusion restriction) in the same region, raising suspicion of a malignant uterine neoplasm. (C) Axial T1-weighted MRI demonstrating a large heterogeneous pelvic mass with predominant T1 hyperintensity, suggestive of hemorrhagic/proteinaceous content. The marked T1 hyperintensity, together with diffusion restriction on DWI/ADC sequences, contributed to the preoperative radiologic suspicion of malignancy. (D) Coronal T2-weighted MRI of the pelvis demonstrates a markedly distended fluid-filled uterine cavity with homogeneous T2 hyperintense contents. A well-defined hypointense soft-tissue lesion is observed arising from the lower uterine segment/cervical region, causing obstruction of the endometrial cavity. No obvious large complex adnexal mass is identified on this image.

A 45-year-old woman with well-controlled hypertension presented with urinary frequency, dysuria, lower abdominal pain, and progressive abdominal distension for the last six months. There was no abnormal uterine bleeding. Abdominal examination revealed a firm, non-tender pelvic mass corresponding to a 28-30-week-sized uterus. Transabdominal ultrasonography demonstrated a markedly enlarged uterus with an apparent fluid-filled cavity, raising suspicion of hematometra or an infected intrauterine collection. Cervical dilatation was attempted but was unsuccessful, with a provisional diagnosis of an organized collection for further investigation. Her magnetic resonance imaging revealed an enlarged uterus measuring 17.3 × 11.9 × 13.5 cm with approximately 63 cc of intracavitary fluid that was hyperintense on T1- and T2-weighted sequences, suggestive of blood products (Figure 1).

**Figure 2:**
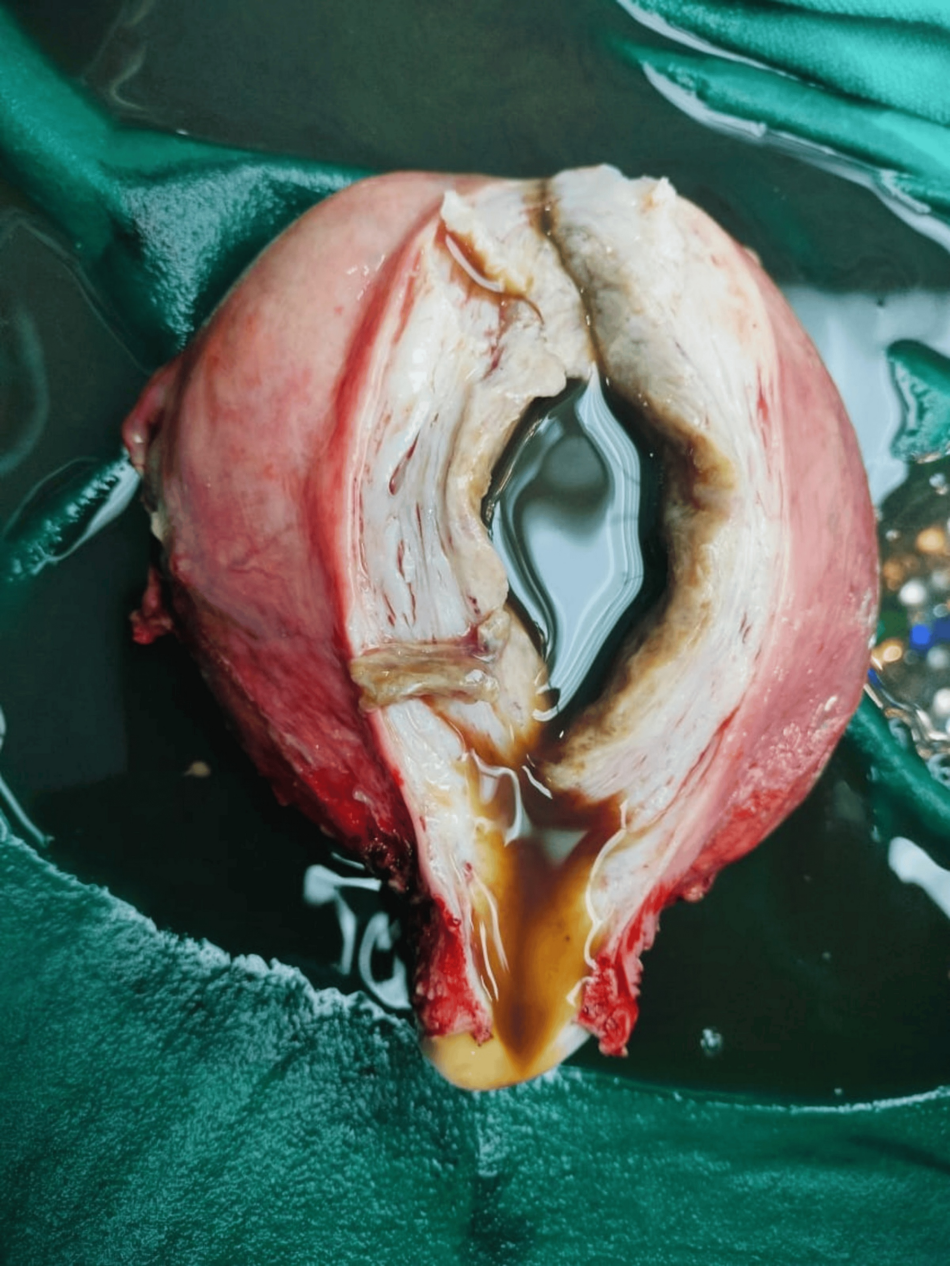
Gross specimen of the uterus (cut section) showing a large degenerating leiomyoma with a central cystic/hemorrhagic collection mimicking hematometra.

The imaging appearance raised concern for malignancy. Given the patient’s symptoms, uterine size, and diagnostic uncertainty, a total hysterectomy was performed 12 days after admission (Figure 2).

**Figure 3:**
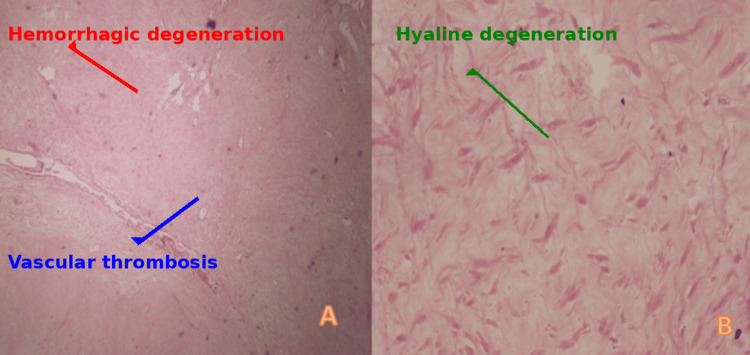
(A) Low-power photomicrograph shows a well-circumscribed smooth muscle tumor with extensive areas of hyaline degeneration, appearing as acellular, homogenous eosinophilic material replacing the smooth muscle bundles (H&E, low power). (B) The section shows vascular compromise with congested and thrombosed vessels at the periphery of the infarcted area, supporting ischemic necrosis as the underlying mechanism (H&E, intermediate power). H&E, hematoxylin and eosin.

Intraoperative frozen section demonstrated necrotic smooth muscle tissue without malignancy. Final histopathology confirmed a uterine leiomyoma with extensive hyaline degeneration and intratumoral hemorrhagic infarction (Figure 3).

## Discussion

Uterine leiomyomas are common benign smooth muscle tumors that may undergo various secondary degenerative changes due to an inadequate vascular supply compared with tumor growth [2]. Degeneration is reported in approximately 5%-10% of leiomyomas [2]. Although leiomyomas are usually benign, extensive degeneration can significantly alter their clinical and radiological appearance, creating diagnostic challenges.

Hemorrhagic degeneration, also known as red degeneration, occurs due to venous obstruction, thrombosis, and subsequent infarction within the leiomyoma. It is classically associated with pregnancy, particularly during the second and third trimesters; however, its occurrence in non-pregnant women is uncommon and may lead to diagnostic uncertainty [4,8]. In the present case, the patient was a perimenopausal woman without pregnancy-related risk factors, making the diagnosis particularly challenging. The absence of abnormal uterine bleeding with the pressure symptoms further contributed to the atypical clinical picture.

The large size of the degenerating leiomyoma resulted in significant mass effect, producing urinary frequency and dysuria due to compression of adjacent pelvic structures. Similar pressure-related symptoms are frequently observed with large fibroids; however, the unusual imaging appearance in this case suggested an intrauterine collection rather than a typical solid fibroid. Ultrasonography demonstrated an apparent fluid-filled uterine cavity, raising suspicion of hematometra or an infected collection. Failure of cervical drainage further increased diagnostic uncertainty.

Magnetic resonance imaging plays an important role in characterizing uterine masses and differentiating benign from malignant pathology. In hemorrhagic degeneration, areas of high signal intensity on T1-weighted images represent blood products, particularly methemoglobin, while T2 signal characteristics may vary depending on the stage of hemorrhage and associated degenerative changes [3,6]. In this case, the presence of T1-hyperintense intracavitary material mimicked retained blood products and raised concern for possible malignancy. Degenerated leiomyomas may demonstrate atypical MRI features overlapping with leiomyosarcoma and other pelvic pathologies.

The differential diagnosis of an enlarged uterus containing apparent intracavitary fluid includes hematometra, pyometra, endometrial malignancy, and degenerating fibroids [6]. In such situations, imaging alone may not provide a definitive diagnosis. Histopathological evaluation remains the gold standard for confirmation and is essential to distinguish benign degenerative changes from malignant transformation [9,10]. In the present case, frozen section examination excluded malignancy, while final histopathology revealed an infarcted leiomyoma with extensive hyaline degeneration and intratumoral hemorrhage.

This case highlights an uncommon presentation of hemorrhagic infarction within a uterine leiomyoma mimicking hematometra in a non-pregnant woman. Awareness of such atypical degenerative patterns is important to avoid misinterpretation of imaging findings and to frame a appropriate surgical planning. A multidisciplinary approach involving clinical examination, radiological and histopathological evaluation remains essential in cases of rapidly enlarging or atypically appearing uterine masses.

## Conclusions

Hemorrhagic degeneration of uterine leiomyoma in non-pregnant women is rare and may closely mimic hematometra or malignancy on imaging. Awareness of this entity and careful clinic-radiological correlation are essential. Histopathology remains crucial for definitive diagnosis and appropriate management.
